# Effect of Three Commercially Available Die Hardening Agents on the Compressive Strength and Surface Hardness of Type IV Dental Stone: An In Vitro Study

**DOI:** 10.7759/cureus.115638

**Published:** 2026-09-02

**Authors:** Lakshmi S Akella, Sidrah Anum, Samved P Sindkar, Dhwani Patel, Rushieswar Chirumamilla, Devi P Nunna

**Affiliations:** 1 Department of Prosthodontics, Gandhi Institute of Technology and Management (GITAM) Dental College and Hospital, Visakhapatnam, IND; 2 Department of Dental Surgery, de' Montmorency College Of Dentistry, Lahore, PAK; 3 Department of Dental Surgery, Maratha Mandal's Nathajirao G. Halgekar Institute of Dental Sciences and Research Centre, Belagavi, IND; 4 Department of Dental Surgery, Government Dental College and Hospital, Ahmedabad, IND; 5 Department of Dental Surgery, Sri Venkata Sai (SVS) Institute of Dental Sciences, Mahabubnagar, IND; 6 Department of Dental Surgery, Mamata Dental College, Khammam, IND

**Keywords:** compressive strength, dental stone, gypsum, prosthodontics, surface properties

## Abstract

Introduction: Type IV dental stone is the material of choice for fabricating working dies in fixed prosthodontics because of its high strength and dimensional accuracy. However, repeated laboratory manipulation may reduce its surface integrity through abrasion and marginal deterioration. Limited evidence exists regarding the combined effect of various die hardeners and drying methods on the mechanical properties of Type IV dental stone. Therefore, the present in vitro study aimed to evaluate the effects of three commercially available die hardeners, used with two different drying protocols, on the compressive strength and surface hardness of Type IV dental stone. Materials and methods: This in vitro experimental study was conducted on 80 standardized Type IV dental stone specimens that were prepared and divided into four experimental groups according to the die hardener used: cyanoacrylate, resin-based, colloidal silica-based, and untreated control. Each group was further subdivided into two drying protocols: 24-hour air drying and one-hour air drying followed by microwave drying. Compressive strength and surface hardness were evaluated using standardized laboratory testing procedures. Data were analyzed using two-way analysis of variance, Tukey's post hoc test, and an independent-samples t-test, with statistical significance set at p < 0.05. Results: The cyanoacrylate die hardener combined with microwave drying demonstrated the highest mean surface hardness (87.6 ± 3.8 Vickers hardness number (VHN)) and compressive strength (73.2 ± 4.1 megapascals (MPa)), whereas the untreated control with 24-hour air drying showed the lowest values (60.5 ± 4.9 VHN and 52.4 ± 5.0 MPa, respectively). Both the type of die hardener and the drying protocol significantly influenced surface hardness (F = 78.45 and 32.78, respectively; p < 0.001) and compressive strength (F = 68.42 and 33.10, respectively; p < 0.001). Microwave drying increased surface hardness by 4.3-5.3 VHN across the die hardener groups and increased compressive strength by 4.5-4.7 MPa in the cyanoacrylate- and resin-treated groups; these increases were statistically significant for the cyanoacrylate and resin groups compared with conventional air drying. Cyanoacrylate produced significantly greater improvements than the colloidal silica-based hardener for both surface hardness (mean difference = 13.75 VHN, p < 0.001) and compressive strength (mean difference = 10.45 MPa, p < 0.001). Conclusions: The application of die hardener significantly improved the mechanical properties of Type IV dental stone. Among the tested materials, the cyanoacrylate-based die hardener produced the greatest enhancement, particularly when combined with microwave drying. This combination may enhance the mechanical properties of Type IV dental stone used for working dies in fixed prosthodontic procedures.

## Introduction

Type IV dental stone is one of the most widely used gypsum products in fixed prosthodontics because of its high strength, dimensional accuracy, and ability to reproduce fine surface details. It serves as the foundation for fabricating crowns, fixed partial dentures, implant-supported prostheses, and other indirect restorations where precision is essential [1,2]. Despite its favorable mechanical properties, the surface of Type IV dental stone remains susceptible to abrasion, chipping, and wear during routine laboratory procedures, such as wax pattern fabrication, margin finishing, and repeated die manipulation [1]. These surface alterations may compromise the accuracy of the final restoration, resulting in poor marginal adaptation and reduced clinical longevity.

To overcome these limitations, die hardeners have been introduced to reinforce the surface characteristics of gypsum dies [3]. These materials penetrate the porous stone surface, improving abrasion resistance by increasing surface hardness and reducing porosity. Commercially available die hardeners are formulated using different chemistries, including cyanoacrylate, resin-based formulations, and colloidal silica, each exhibiting distinct mechanisms of penetration and surface reinforcement [4]. In addition to the die hardener itself, the drying protocol employed before application may influence the effectiveness of surface penetration and the resulting mechanical properties [5]. Conventional air drying is routinely practiced, whereas microwave drying has been proposed as a faster alternative that can enhance moisture removal and improve gypsum strength [6]. However, evidence comparing the combined influence of different die hardeners and drying protocols on the mechanical and surface characteristics of Type IV dental stone remains limited and inconclusive.

Therefore, the aim of this in vitro study was to evaluate the effects of three commercially available die hardeners, used with two different drying protocols, on the compressive strength and surface hardness of Type IV dental stone. The objectives of the present study were to evaluate the effect of three commercially available die hardening agents, namely, cyanoacrylate-based, resin-based, and colloidal silica-based agents, on the compressive strength (primary outcome) of Type IV dental stone compared with untreated specimens; to determine the effect of two different drying protocols, namely, 24-hour conventional air drying and one hour of air drying followed by microwave drying at 600 W for 10 minutes, on compressive strength; to compare their effects on surface hardness; and to identify the combination of die hardener and drying protocol associated with the most favorable mechanical and surface properties. It was hypothesized that the application of die hardening agents would improve the mechanical and surface properties of Type IV dental stone compared with untreated specimens and that a protocol involving one hour of air drying followed by microwave drying would yield greater improvement than conventional 24-hour air drying.

## Materials and methods

Study design and setting

This in vitro experimental study was conducted in the Department of Prosthodontics, GITAM Dental College and Hospital, Visakhapatnam, India, between January 2018 and May 2018. All laboratory procedures were carried out under standardized environmental conditions to minimize experimental variability. Since the study exclusively involved commercially available dental materials and laboratory testing, without human participants, tissues, biological samples, or patient records, ethical approval was not required in accordance with institutional guidelines and international recommendations for laboratory-based in vitro studies.

Sample size calculation

The required sample size was determined a priori using G*Power software version 3.1.9.7 (Heinrich-Heine-Universität Düsseldorf, Düsseldorf, Germany). Based on a previous investigation evaluating the effect of die hardeners on the mechanical properties of Type IV dental stone, a moderate-to-large effect size (Cohen's f = 0.35) was considered for the primary outcome measures [3]. Assuming a significance level (α) of 0.05, statistical power (1−β) of 80%, and a two-way analysis of variance (ANOVA) design, the minimum required sample size was calculated to be 64 specimens. To compensate for potential specimen loss due to fabrication defects, air voids, handling damage, or testing-related fractures, the sample size was increased by 25%, yielding a final sample size of 80 specimens.

Materials and specimen preparation

Fujirock EP Type IV dental stone (GC Corporation, Tokyo, Japan) was used to fabricate all specimens. Three commercially available die hardeners, namely, a cyanoacrylate-based die hardener (Zhermack SpA, Badia Polesine, Rovigo, Italy), a resin-based die hardener (Yeti Dentalprodukte GmbH, Engen, Germany), and a colloidal silica-based die hardener (Kerr Corporation, Orange, California, USA), were evaluated. Additional materials included distilled water, standardized silicone molds, disposable microbrushes, a digital weighing balance, a laboratory vibrator, and a Multivac 4 vacuum mixer (Degussa AG, Hanau, Germany). The dental stone was prepared according to the manufacturer’s recommended powder-to-water ratio under controlled laboratory conditions (23 ± 2°C and 50 ± 10% relative humidity). The powder and distilled water were mixed under vacuum using a vacuum mixer for approximately 30 seconds to minimize air entrapment. The mixed material was poured into standardized silicone molds (2 x 2 x 2 cm) using a laboratory vibrator to eliminate voids and ensure complete mold adaptation. After the initial setting period, all specimens were carefully removed from the molds and stored under laboratory conditions until the assigned drying protocol was completed.

Group allocation and drying protocol

A total of 80 specimens were randomly allocated into four experimental groups (n = 20) according to the die hardener applied. Group I received a cyanoacrylate die hardener, Group II received a resin-based die hardener, Group III received a colloidal silica-based die hardener, and Group IV served as the untreated control without a die hardener. Each experimental group was further divided into two equal subgroups (n = 10) according to the drying protocol. In Subgroup A, specimens were allowed to air dry at room temperature for 24 hours before application of the die hardener. In Subgroup B, specimens were air-dried for one hour and then dried in a microwave oven (Samsung MC28H5135VK, Samsung Electronics, Malaysia; rated output 900 W; 2450 MHz) at 600 W for 10 minutes. Specimens were placed on the rotating turntable without contacting each other or the chamber walls. A glass beaker containing 400 mL of distilled water was placed in the chamber as a heat sink. After irradiation, all specimens were allowed to cool to room temperature before application of the respective die hardener (Figure 1).

**Figure 1:**
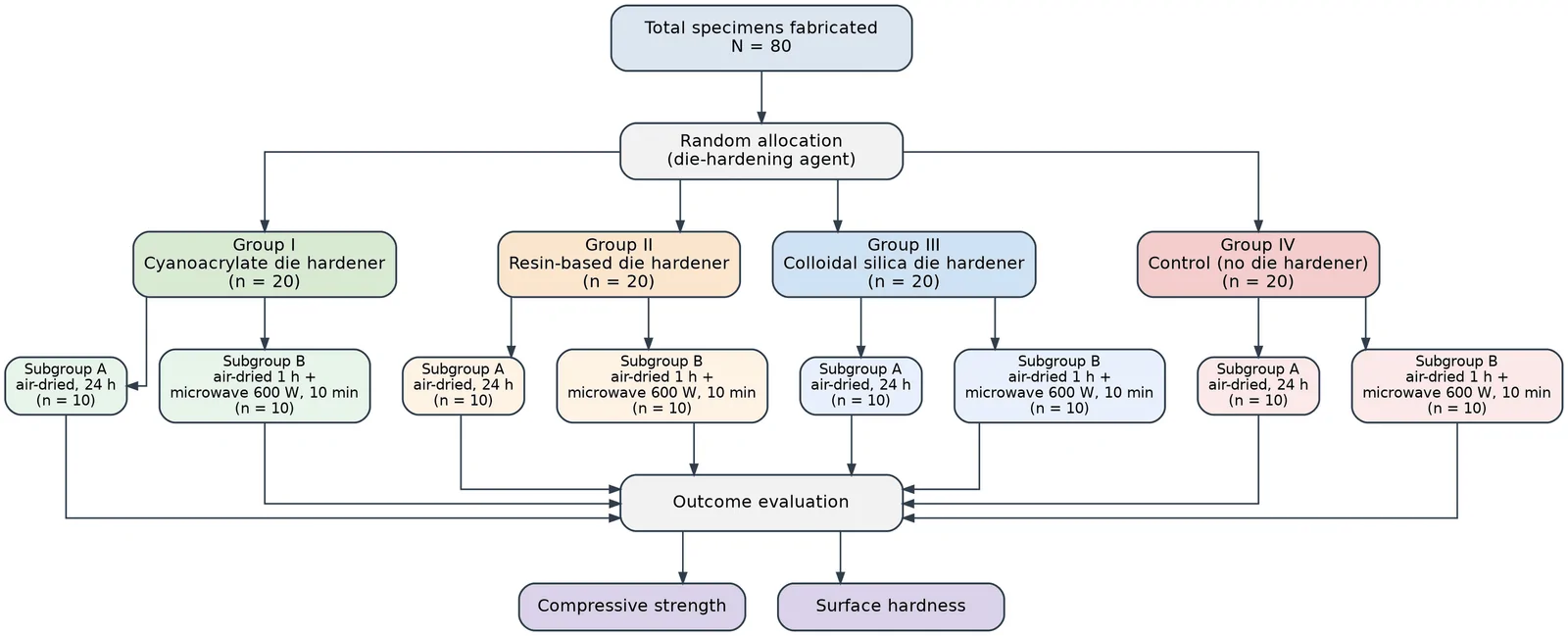
Study group assignment Image created using Canva (Canva Pty Ltd., Australia).

Application of die hardeners

Following completion of the assigned drying protocol, the corresponding die hardener (2 mL per specimen) was uniformly applied to the entire surface of each specimen using disposable microbrushes, according to the manufacturer's recommendations. A single thin coat of the designated die hardener was applied to ensure standardized surface coverage. The treated specimens were allowed to dry completely before mechanical testing. Control specimens underwent identical drying procedures but did not receive any surface treatment.

Compressive strength evaluation

The compressive strength of all specimens was evaluated using a universal testing machine (Instron 3366, Instron Corporation, Norwood, MA, USA). Each specimen was positioned centrally between the compression platens, and a compressive load was applied at a crosshead speed of 1 mm/min until fracture occurred. The maximum load at fracture was automatically recorded by the testing software, and the compressive strength (megapascals (MPa)) was calculated by dividing the maximum load by the cross-sectional area of the specimen.

Surface hardness evaluation

Surface hardness was measured using a Vickers Microhardness Tester (HMV-G Series, Shimadzu Corporation, Kyoto, Japan). A 500 g load was applied for 15 seconds per indentation. Three indentations were made at different locations on each specimen while avoiding the margins and previously indented areas. The average of the three measurements was recorded as the final Vickers hardness number (VHN) for statistical analysis.

Blinding

The investigator responsible for evaluating compressive strength and surface hardness was blinded to the group allocation throughout the testing procedures. Data analysis was performed using coded specimen identifiers to minimize assessment bias.

Statistical analysis

Statistical analysis was performed using SPSS Statistics version 25.0 (IBM Corp. Released 2017. IBM SPSS Statistics for Windows, Version 25.0. Armonk, NY). Data distribution was assessed using the Shapiro-Wilk test. Continuous variables were expressed as mean ± standard deviation (SD) together with 95% confidence intervals (CIs). The effects of the type of die hardener and drying protocol on compressive strength and surface hardness were analyzed using two-way ANOVA. When statistically significant differences were identified, Tukey's honestly significant difference post hoc test was performed for pairwise comparisons. Comparisons between the two drying protocols within each die hardener group were performed using the independent-samples t-test. Effect sizes were reported as partial eta squared (η²), and all statistical tests were two-tailed with the level of statistical significance set at p < 0.05.

## Results

A total of 80 Type IV dental stone specimens were included in the analysis, with 20 specimens allocated to each experimental group and 10 specimens in each drying protocol subgroup. All specimens underwent the experimental procedures, and none were excluded from the final analysis. The evaluated outcomes included compressive strength and surface hardness. The cyanoacrylate die hardener combined with microwave drying (Group IB) demonstrated the highest mean surface hardness (87.6 ± 3.8 VHN) and compressive strength (73.2 ± 4.1 MPa). In contrast, the untreated control group subjected to 24-hour air drying (Group IVA) exhibited the lowest values for both surface hardness (60.5 ± 4.9 VHN) and compressive strength (52.4 ± 5.0 MPa). Across all die hardener groups, the specimens subjected to microwave drying consistently showed higher mean values than those dried under conventional air-drying conditions. The CIs indicated relatively low variability within each experimental subgroup (Table 1).

**Table 1:** Surface hardness and compressive strength according to the experimental groups Data are presented as mean ± SD and 95% CI. VHN: Vickers hardness number, MPa: megapascal, CI: confidence interval, SD: standard deviation

Group	n	Surface hardness (VHN)		Compressive strength (MPa)	
		Mean ± SD	95% CI	Mean ± SD	95% CI
IA (cyanoacrylate + 24 h air drying)	10	82.4 ± 4.2	79.4-85.4	68.7 ± 4.5	65.5-71.9
IB (cyanoacrylate + microwave drying)	10	87.6 ± 3.8	84.9-90.3	73.2 ± 4.1	70.3-76.1
IIA (resin + 24 h air drying)	10	79.8 ± 4.5	76.6-83.0	65.4 ± 4.8	62.0-68.8
IIB (resin + microwave drying)	10	84.1 ± 4.0	81.2-87.0	70.1 ± 4.3	67.0-73.2
IIIA (colloidal silica + 24 h air drying)	10	68.3 ± 5.1	64.7-71.9	58.2 ± 5.2	54.5-61.9
IIIB (colloidal silica + microwave drying)	10	73.6 ± 4.7	70.2-77.0	62.8 ± 4.9	59.3-66.3
IVA (control + 24 h air drying)	10	60.5 ± 4.9	57.0-64.0	52.4 ± 5.0	48.8-56.0
IVB (control + microwave drying)	10	64.2 ± 4.6	60.9-67.5	56.1 ± 4.6	52.8-59.4

Both the type of die hardener and drying protocol significantly influenced the surface hardness and compressive strength (p < 0.001). The die hardener produced the largest effect on both outcome variables, with large effect sizes (partial η² > 0.70). However, the interaction between the die hardener type and the drying protocol was not statistically significant (p > 0.05), indicating that the improvement achieved by microwave drying was consistent regardless of the die hardener used (Table 2).

**Table 2:** Two-way ANOVA for surface hardness and compressive strength Two-way ANOVA was used; *p < 0.05 was considered statistically significant. df: degrees of freedom, η²: partial eta squared, VHN: Vickers hardness number, MPa: megapascals, ANOVA: analysis of variance

Outcome measures	Source	Sum of squares	df	Mean square	F value	p-value	Partial η²
Surface hardness (VHN)	Die hardener	4,826.3	3	1,608.8	78.45	<0.001*	0.752
	Drying protocol	672.4	1	672.4	32.78	<0.001*	0.301
	Interaction (hardener × drying)	124.8	3	41.6	2.03	0.116	0.075
Compressive strength (MPa)	Die hardener	3,215.7	3	1,071.9	68.42	<0.001*	0.740
	Drying protocol	518.4	1	518.4	33.10	<0.001*	0.315
	Interaction (hardener × drying)	98.6	3	32.9	2.10	0.108	0.080

All three die hardeners produced significantly greater hardness than the untreated control (p < 0.001). The cyanoacrylate die hardener demonstrated the highest hardness values and was significantly superior to the colloidal silica-based hardener (p < 0.001). In contrast, no statistically significant difference was observed between the cyanoacrylate and resin-based die hardeners (p = 0.082). The resin-based die hardener also produced significantly greater hardness than the colloidal silica-based material (p < 0.001), as shown in Table 3.

**Table 3:** Tukey's post hoc multiple comparisons for surface hardness Tukey's honestly significant difference post hoc test was used; *p < 0.05 was considered statistically significant. SE: standard error, CI: confidence interval, VHN: Vickers hardness number

Comparison	Mean difference (VHN)	SE	95% CI	Adjusted p-value
Cyanoacrylate vs resin	2.95	1.12	-0.28-6.18	0.082
Cyanoacrylate vs colloidal silica	13.75	1.12	10.52-16.98	<0.001*
Cyanoacrylate vs control	22.50	1.12	19.27-25.73	<0.001*
Resin vs colloidal silica	10.80	1.12	7.57-14.03	<0.001*
Resin vs control	19.55	1.12	16.32-22.78	<0.001*
Colloidal silica vs control	8.75	1.12	5.52-11.98	<0.001*

The cyanoacrylate die hardener exhibited significantly greater compressive strength than the resin-based, colloidal silica-based, and untreated control groups (p < 0.05). The resin-based die hardener also exhibited significantly higher compressive strength than the colloidal silica and control groups (p < 0.001). All die hardeners significantly improved the compressive strength of the specimens compared to the untreated specimens (Table 4).

**Table 4:** Tukey's post hoc multiple comparisons for compressive strength Tukey's honestly significant difference post hoc test was used; *p < 0.05 was considered statistically significant. SE: standard error, CI: confidence interval, MPa: megapascals

Comparison	Mean difference (MPa)	SE	95% CI	Adjusted p-value
Cyanoacrylate vs resin	3.20	0.99	0.27-6.13	0.031*
Cyanoacrylate vs colloidal silica	10.45	0.99	7.52-13.38	<0.001*
Cyanoacrylate vs control	16.70	0.99	13.77-19.63	<0.001*
Resin vs colloidal silica	7.25	0.99	4.32-10.18	<0.001*
Resin vs control	13.50	0.99	10.57-16.43	<0.001*
Colloidal silica vs control	6.25	0.99	3.32-9.18	<0.001*

Microwave drying significantly increased the surface hardness in the cyanoacrylate (p = 0.009), resin-based (p = 0.034), and colloidal silica (p = 0.024) groups. In contrast, the increase observed in the untreated control specimens was not statistically significant (p = 0.094). Similarly, microwave drying significantly improved the compressive strength of specimens treated with cyanoacrylate (p = 0.029) and resin-based die hardeners (p = 0.031). Although the colloidal silica and control groups demonstrated numerically higher compressive strength after microwave drying, these differences did not reach statistical significance (p = 0.054 and p = 0.098, respectively) (Table 5).

**Table 5:** Comparison of drying protocols within each die hardener group Independent-samples t-test was used; *p < 0.05 was considered statistically significant. SD: standard deviation, VHN: Vickers hardness number, MPa: megapascals

Outcome measure	Die hardener (n = 20)	Air drying, mean ± SD, (n = 10)	Microwave drying, mean ± SD, (n = 10)	Mean difference	t-value	p-value	Effect size (Cohen's d)
Surface hardness (VHN), (n = 80)	Cyanoacrylate	82.4 ± 4.2	87.6 ± 3.8	5.2	2.94	0.009*	1.30
	Resin	79.8 ± 4.5	84.1 ± 4.0	4.3	2.29	0.034*	1.01
	Colloidal silica	68.3 ± 5.1	73.6 ± 4.7	5.3	2.46	0.024*	1.08
	Control	60.5 ± 4.9	64.2 ± 4.6	3.7	1.77	0.094	0.78
Compressive strength (MPa), (n = 80)	Cyanoacrylate	68.7 ± 4.5	73.2 ± 4.1	4.5	2.37	0.029*	1.05
	Resin	65.4 ± 4.8	70.1 ± 4.3	4.7	2.34	0.031*	1.03
	Colloidal silica	58.2 ± 5.2	62.8 ± 4.9	4.6	2.06	0.054	0.91
	Control	52.4 ± 5.0	56.1 ± 4.6	3.7	1.75	0.098	0.77

Comparison of the two drying protocols showed that the one-hour air-drying followed by microwave drying protocol significantly increased surface hardness in the cyanoacrylate, resin-based, and colloidal silica groups compared with 24-hour conventional air drying. Similarly, compressive strength was significantly higher with the combined drying protocol in the cyanoacrylate and resin-based groups. These findings indicate differences between the two drying protocols; however, the independent contributions of drying duration and microwave exposure cannot be separated in the present experimental design.

## Discussion

The present study compared two drying protocols: conventional 24-hour air drying and one hour of air drying followed by microwave drying at 600 W for 10 minutes. The results demonstrated that specimens subjected to the latter protocol generally exhibited higher surface hardness and compressive strength than those subjected to conventional 24-hour air drying. Because the two protocols differed in both drying duration and drying method, the observed differences should be interpreted as the effect of the combined drying protocols rather than being attributed exclusively to microwave exposure.

Type IV dental stone is widely used to fabricate dies in fixed prosthodontics because of its dimensional stability and strength [1,2]. Nevertheless, repeated manipulation during wax pattern fabrication and restoration finishing can result in abrasion and loss of marginal detail. Therefore, improving the surface characteristics of gypsum dies is essential for maintaining the accuracy of the restoration [3]. The present findings indicate that surface treatment with die hardeners substantially enhances the resistance of gypsum dies to mechanical deterioration.

The superior performance of the cyanoacrylate die hardener observed in this study may be attributed to its low viscosity and excellent penetration into the micropores of the gypsum matrix. The findings of the present study are consistent with those reported by Lindquist et al. [7], who showed that Permabond 910 cyanoacrylate produced the greatest reduction in material loss compared with untreated specimens, indicating superior reinforcement of the gypsum surface. The authors suggested that the effectiveness of cyanoacrylate results from its ability to penetrate surface porosities and form a polymerized protective layer that resists abrasion during laboratory procedures. These observations support the present findings, which indicate that the cyanoacrylate-based die hardener produced the highest surface hardness and compressive strength among all evaluated materials. Furthermore, the authors reported that surface hardeners significantly reduced water sorption in selected gypsum materials, suggesting that forming a sealed surface layer reduces moisture uptake and improves the durability of dental stone dies [7]. The improved mechanical properties observed in the present study may similarly be attributed to reduced surface porosity and enhanced crystal reinforcement following the application of a die hardener.

The resin-based die hardener also significantly improved the evaluated properties compared with the untreated control, although the improvements were slightly lower than those achieved with cyanoacrylate. Resin-based materials primarily act by forming a protective surface coating that partially penetrates the gypsum surface while sealing the superficial pores. Although this coating effectively enhances hardness and compressive resistance, its penetration depth is generally lower than that achieved with cyanoacrylate, which may explain the comparatively lower mechanical properties observed in this study.

The findings of the present study are in agreement with those reported by Lindquist et al. [4], who evaluated the abrasion resistance of conventional Type IV gypsum and resin-impregnated Type IV gypsum. The authors observed that Type IV gypsum materials exhibited significantly greater abrasion resistance than conventional gypsum, confirming the superior mechanical properties of high-strength dental stone for die fabrication. Although no statistically significant difference was found between the conventional and resin-impregnated Type IV stones, the resin-impregnated material consistently demonstrated the least material loss during abrasion testing, suggesting that polymer reinforcement may provide additional protection against surface wear.

The colloidal silica-based die hardener showed the least improvement in surface hardness and compressive strength among the evaluated die hardeners. However, it still achieved significantly greater surface hardness and compressive strength than the untreated control. The comparatively lower enhancement may be attributed to the differences in the surface reinforcement mechanisms of the die hardeners. Unlike cyanoacrylate, which polymerizes after penetrating the surface porosities, colloidal silica primarily deposits silica particles on and within the superficial surface of the gypsum, thereby providing comparatively limited reinforcement of the underlying gypsum matrix. Similar observations regarding the differences in the effectiveness of various die hardeners have been reported by Harris et al. [3] and Lindquist et al. [4], who demonstrated that improvements in the mechanical properties of gypsum dies depend on the specific hardener formulation and its interaction with the gypsum material rather than the application of a surface coating alone.

Microwave drying significantly enhanced both surface hardness and compressive strength in most experimental groups. This finding can be explained by the rapid and uniform removal of residual free water from the gypsum matrix. Excess moisture remaining after setting increases porosity and reduces crystal packing density, thereby compromising the material's strength [8]. Microwave energy accelerates water evaporation while promoting closer approximation of interlocking calcium sulfate dihydrate crystals, resulting in a denser and mechanically stronger structure. Similar findings have been reported in previous investigations evaluating microwave drying of gypsum products, where microwave treatment reduced residual moisture and significantly increased compressive strength compared with conventional air drying [8-11].

In contrast, Silva et al. [12] evaluated the influence of microwave and conventional room-temperature drying on the physical properties of three commercially available Type IV dental stone materials. They reported that microwave drying produced compressive strength comparable to specimens dried conventionally for 24 hours in Elite Rock and Durone IV. In contrast, Shera Premium exhibited values similar to those of specimens dried for seven days at room temperature. The authors also observed that microwave drying did not adversely affect linear dimensional stability or detail reproduction.

Interestingly, the interaction between the type of die hardener and drying protocol was not statistically significant. This indicates that microwave drying enhanced the properties of all groups in a relatively similar manner and that the beneficial effects of the die hardeners were largely independent of the drying technique employed. From a materials science perspective, this suggests that moisture removal and surface reinforcement are two complementary yet independent mechanisms that contribute to the improved mechanical properties of gypsum dies.

The findings of the present study are consistent with the available literature indicating that surface treatment improves the durability of gypsum dies [13]. Harris et al. [3] reported significant improvements in surface hardness after applying commercially available die hardeners. At the same time, several subsequent investigations have confirmed that both polymer-based and resin-based surface treatments reduce abrasion and enhance the mechanical stability of Type IV dental stone [6,7]. The present study further extends these observations by simultaneously evaluating multiple die hardeners under different drying protocols and demonstrating the additional benefit of microwave drying.

These findings may have implications for prosthodontic practice and dental laboratory procedures. Improved surface hardness and compressive strength may contribute to greater resistance to mechanical deterioration during laboratory handling and may help preserve the integrity of working dies. Based on the present findings, cyanoacrylate die hardener combined with microwave drying appears to be the most effective protocol for enhancing the mechanical properties of Type IV dental stone and may contribute to improved laboratory efficiency and better restoration quality.

The present study has certain limitations. Because this is an in vitro investigation, laboratory conditions cannot fully replicate the complex clinical and laboratory environment encountered in routine prosthodontic procedures. Only three commercially available die hardeners and two drying protocols were evaluated, and the long-term durability of the treated dies under repeated manipulation was not investigated. In addition, other clinically relevant properties such as dimensional accuracy, abrasion resistance after repeated use, detail reproduction, and compatibility with impression materials and die spacers were not assessed. The comparisons between drying protocols were predefined within each die hardener group based on the primary experimental objective; therefore, independent-samples t-tests were used for these planned within-group comparisons. Nevertheless, the potential for inflation of type I error due to multiple comparisons should be considered when interpreting these secondary within-group comparisons. Future studies incorporating simulated clinical conditions, long-term aging, and evaluation of additional commercially available products are recommended to further validate the present findings.

## Conclusions

Within the limitations of this in vitro study, the application of die hardening agents significantly improved the compressive strength and surface hardness of Type IV dental stone compared with untreated specimens. Among the evaluated materials, the cyanoacrylate-based die hardener demonstrated the greatest improvement. The protocol, consisting of one hour of air drying followed by microwave drying, was associated with a further enhancement of these mechanical properties compared with conventional 24-hour air drying. However, because the two protocols differed in both drying duration and method, the observed improvement cannot be attributed to microwave exposure alone. Further studies are warranted to evaluate abrasion resistance, dimensional accuracy, long-term durability, and clinical performance under simulated clinical conditions.
